# Eight Months of Serological Follow-Up of Anti-SARS-CoV-2 Antibodies in France: A Study among an Adult Population

**DOI:** 10.3390/ijerph192215257

**Published:** 2022-11-18

**Authors:** Dorine Decarreaux, Julie Sevila, Shirley Masse, Lisandru Capai, Toscane Fourié, Paola Mariela Saba Villarroel, Abdennour Amroun, Elif Nurtop, Matthieu Vareille, Thierry Blanchon, Xavier de Lamballerie, Remi Charrel, Alessandra Falchi

**Affiliations:** 1 Laboratoire de Virologie, Université de Corse Pascal Paoli, UR7310 Bioscope, 20250 Corte, France; 2 INSERM, Sorbonne Université, Institut Pierre Louis d’Epidémiologie et de Santé Publique, IPLESP, 75012 Paris, France; 3 Unité des Virus émergents, Aix Marseille University, IRD 190, INSERM U1207, IHU Méditerranée Infection, 13005 Marseille, France

**Keywords:** COVID-19, seroconversion, long-lasting immunity, antibodies, vaccination

## Abstract

Background: Uncertainties remain regarding the nature and durability of the humoral immune response to severe acute respiratory syndrome coronavirus-2 (SARS-CoV-2). Aim: This study investigated immunoglobulin G response and neutralizing activity to evaluate the mean antibody concentrations and response duration induced by each vaccination regimen in a French adult population. Methods: A study including blood sampling and questionnaires was carried out from November 2020 to July 2021 with three separate follow-up phases. Spike proteins and neutralizing antibodies were quantified using ELISA and a virus-neutralization test. Results: Overall, 295 participants were included. Seroprevalences were 11.5% (n = 34), 10.5% (n = 31), and 68.1% (n = 201) in phases 1, 2, and 3, respectively. Importantly, 5.8% (n = 17) of participants lost their natural antibodies. Antibody response of participants with only a prior infection was 88.2 BAU/mL, significantly lower than those vaccinated, which was 1909.3 BAU/mL (*p* = 0.04). Moreover, the antibody response of vaccinated participants with a prior infection was higher (3593.8 BAU/mL) than those vaccinated without prior infection (3402.9 BAU/mL) (*p* = 0.78). Vaccinated participants with or without prior infection had a higher seroneutralization rate (91.0%) than those unvaccinated with prior infection (65.0%). Conclusion: These results demonstrated that single infection does not confer effective protection against SARS-CoV-2.

## 1. Introduction

The emergence of the coronavirus disease 2019 (COVID-19) has caused an unprecedented global public health crisis [1]. Because of its novelty, the continued and rapid spread of the virus worldwide, vaccines were developed in less than one year. In France, the first vaccination campaigns were implemented on 27 December 2020, initially for people living in nursing homes and healthcare workers in contact with patients, and then gradually to the entire population aged 12 years and older from June 2021 [2].

SARS-CoV-2 infection and vaccination can elicit detectable humoral responses, including the production of antibodies against the virus spike protein (S), nucleoprotein (N), and neutralizing antibodies [3,4,5,6]. However, many uncertainties regarding the immune response against SARS-CoV-2 and the duration of immunity have been raised.

Investigations into the time course of antibody levels after infection have yielded conflicting results so far. Previous studies have suggested that antibodies to SARS-CoV-2 remain stable for 4–6 months [7,8,9,10], while others have demonstrated permanence of seropositivity for up to one year, but with decreasing antibody levels [11,12]. Indeed, studies have shown a progressive decrease in antibodies induced by natural infection [13,14] or vaccination, including neutralizing antibodies [15,16,17].

In the current context, we decided to set up a study using an eight-month serological follow-up in three steps. The study was carried out in France in an adult population between the second and fourth epidemic waves of COVID-19. Our main objective was to characterize the humoral status of participants in relation to infection and/or vaccination. In this follow-up study, we were able to follow the evolution of antibody levels of the participants from the University of Corsica (France) who had tested positive in a previous article [18].

The results of this study may contribute to the understanding of the immune response in order to provide information on the prevention and control of COVID-19, as well as vaccination strategies and the prevention of future outbreaks.

## 2. Materials and Methods

### 2.1. Timeline

**Figure 1:**
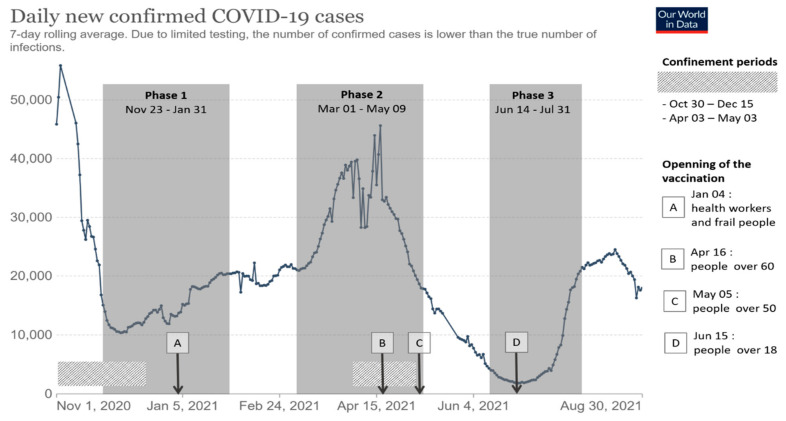
Timeline of the French COVID-19 epidemic during the study period.

The study was conducted between the second and fourth epidemic waves of COVID-19 (August 2020 to August 2021) in France (Figure 1).

The first phase of the study took place between the second and third epidemic waves of COVID-19, whereas the second phase of the study took place during the third epidemic wave of COVID-19 and the third phase of the study took place during the fourth epidemic wave of COVID-19.

The second wave of COVID-19, during which a lockdown was instituted in October 2021, occurred in France from 24 August to 21 December 2020. At this time, the majority of enterprises were allowed to remain open, with the exception of establishments that were open to the public (nonessential stores, bars, restaurants, entertainment venues, etc.). Universities continued to operate normally from the beginning of September 2020 through 18 December 2020, with the exception of the two-week autumn break. Universities were able to remain open via the implementation of strict health protocols (wearing masks, spacing classes, physical distancing, contact tracing, testing, and quarantine). During this second wave, students at the University of Corsica could attend in-person classes once or twice per week while staff members were on-site at the university campus, with possibilities for telecommuting (at least once a week).

The development of the first vaccines against COVID-19 has experienced great progress since November 2020. The vaccination campaign started at the end of December 2020 in France according to an order of priority: (i) elderly people in EHPAD, people with comorbidities, and caregivers; (ii) people over 60 years of age; (iii) people over 50 years of age; and, finally, (iv) people over 18 years of age as of 15 June 2021. Despite this deployment of vaccination, a third wave of COVID-19 affected France from 4 January to 5 July, 2021, particularly because of the appearance of many variants of the virus. By the end of 2020, the Alpha variant appeared in France and became the predominant variant in March 2021. During the first half of 2021, the Beta and Gamma variants also circulated in this country, albeit to a lesser extent. This third wave led to an overload of hospital services and generated the implementation of new restrictions, such as the establishment of a daily curfew from 6:00 p.m. to 6:00 a.m. from 16 January 2021. After opting for localized containment measures by territory, the government finally extended the third containment to the whole country on 3 April 2021, with lighter restrictions than those implemented during the previous containments (more businesses were allowed to stay open, and travel without attestation up to 10 km around the home was permitted). Schools, colleges, and high schools had to close, whereas universities were spared. Students could still attend classes 1 day per week while maintaining barrier gestures. On 3 May 2021, the containment ended, as did the limitation of movement with authorization to 10 km.

On 21 July 2021, France entered its fourth epidemic wave of COVID-19. In May 2021, the Delta variant replaced the previous variants and became predominant in July 2021; it accounted for more than 99% of the variants in circulation in France as of August 2021. The acceleration of the vaccination campaign has allowed the government to set up a progressive reopening schedule for businesses and establishments that receive the public (bars, restaurants, museums, cinemas, and parks), with attendance limits. As of 30 June 2021, an easing of the restrictions was implemented, with the end of the curfew and the limits of gauge in the places that receive the public (measure subjected to the local health situation). Upon presentation of a health pass, access to events with more than 1000 people (either indoors or outdoors) became possible. As a result, higher education institutions will be able to accommodate 100% of their capacity in the classroom at the beginning of the 2021 academic year (Figure 1).

### 2.2. Study Design and Participants

**Figure 2:**
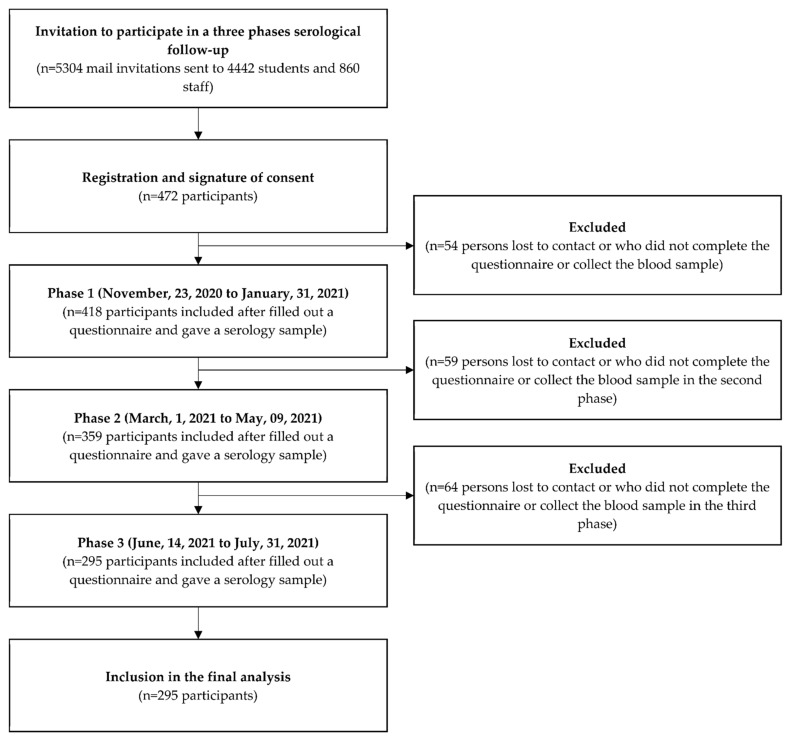
Flowchart of the study population.

The CRONOS-COVID project in Corsica, France is a university-population-based study (staff and students) aimed to investigate the seroprevalence and immune durability of anti-SARS-CoV-2 antibodies from 23 November 2020 to 31 July 2021. Previously enrolled study participants [18] were asked to return for a second and third phase. In total, 3 collection points were set up. The first phase took place from 23 November 2020 to 31 January 2021. The second phase took place from 1 March to 9 May 2021. Finally, the third phase took place from 14 June to 31 July 2021 (see §Timeline). This study included the voluntary participation of the staff and students of the University of Corsica, Corte, France. A total of 295 participants were included in the study after signing a consent form, and took part in all 3 phases of the study by providing a blood sample and completing a questionnaire at each phase. The flowchart presented in Figure 2 describes the sample-inclusion process of the final analysis.

### 2.3. Outcomes

The main outcome of this study was the evaluation of the evolution of anti-SARS-CoV-2 antibodies among the same adult population according to their immune status, i.e., history of COVID-19 infection, vaccination, and/or both. The first outcome was the estimation of the seroprevalence of anti-SARS-CoV-2 over 8 months through 3 phases. The secondary outcome was the measurement of the evolution of antibody levels and the presence of neutralizing antibodies in the seropositive population as a function of the immune status (history of COVID-19 infection, vaccination, and/or both).

### 2.4. Serological Analysis

Capillary blood samples were obtained from the university population using a safety lancet on the finger of participants and collected into coagulation activator serum tubes. Subsequently, the tubes were centrifuged for 15 min at 6000 rpm, and the serum was stored at −20 °C until analysis. The IgG antibodies to the SARS-CoV-2 receptor binding domain (RBD) of the Spike protein were evaluated using Euroimmun’s semiquantitative anti-SARS-CoV-2 (IgG) ELISA (reference: EI 2606-9601 G; EUROIMMUN, Bussy-Saint-Martin, France), with a specificity of 99.8% and a sensitivity of 90.3%. Serum samples were analyzed in duplicate. According to the manufacturer’s instructions, the results were evaluated by calculating the ratio of the optical density of the patient sample to the optical density of the calibrator. A test was considered borderline if the ratio was between 0.8 and 1.1, and positive when the results indicated an optical density ratio ≥ 1.1. Then, for all samples with a positive ELISA-Spike (ELISA-S) (ratio > 0.8) and if sufficient serum was available, IgG antibodies were quantified using the QuantiVac (IgG) kit (reference: El 2606-9601-10 G, EUROIMMUN, Bussy-Saint-Martin, France). The quantification of IgG was performed using a 6-point standard curve covering a range of 1 to 120 relative units (RU)/mL. The results in RU/mL were converted to standardized binding antibody units (BAU)/mL by multiplication with a factor of 3.2. According to the manufacturer’s instructions, a result of <25.6 BAU/mL was considered negative and a result of ≥35.2 BAU/mL was considered positive. In all ELISA-S-positive samples, the presence of neutralizing antibodies was assessed using a virus-neutralization test (VNT), as described previously [19]. Vero E6 cells cultivated in 96-well microplates, 100 tissue culture infectious doses (TCID50) of the SARS-CoV-2 strain BavPat1 (courtesy of Professor Drosten, Berlin, Germany), and serial dilutions of serum (1:20 to 1:160) were performed. Dilutions associated with a cytopathic effect were considered negative (no neutralization), whereas those without such effect on day 4 after infection were considered positive (complete neutralization). The neutralization titer refers to the highest dilution of positive serum that still afforded neutralization. The specimens with a neutralization titer of 20 were considered positive. The ELISA-S and seroneutralization tests were used during all 3 phases of this study, in order to ensure comparability.

### 2.5. Statistical Analysis

For the descriptive statistical methods, we selected all consenting subjects who participated in all 3 phases of the study by providing a serological test and completing a questionnaire at each phase. The cohort was grouped into 4 categories based on their vaccination status and confirmed history of COVID-19. The first class corresponded to participants who were defined as being “Unvaccinated with no known history of infection”, i.e., participants who were not vaccinated, who had not reported a positive PCR, and who did not have a positive serological result on previous phases. The second category corresponded to participants who were defined as being “Unvaccinated with known history of infection”, i.e., participants who were not vaccinated but reported a positive PCR test and/or a positive ELISA test in the previous phase. The third category corresponded to participants who were defined as being “Vaccinated with no known history of infection”, i.e., participants who were vaccinated with at least 1 dose but reported no COVID-19 history ((i) no positive PCR test or (ii) no positive ELISA test in the previous phase). The fourth category corresponded to participants who were defined as being “Vaccinated with known history of infection”, i.e., participants who were vaccinated with at least 1 dose and reported a history of COVID-19 with (i) a positive PCR test and/or (ii) a positive ELISA test in the previous phase. Standard descriptive statistics were used to present the sociodemographic and clinical characteristics of the university population. Categorial data were presented as numbers and percentages. Numerical variables were presented as the mean or median and range (min–max). Seroprevalences and their 95% confidence intervals (CIs) were estimated for each phase on the total number of participants according to COVID-19 vaccination and confirmed history of COVID-19. Subsequently, antibody levels and seroneutralization results were estimated for participants presenting anti-SARS-CoV-2 IgG antibodies (ELISA-S), i.e., a fraction of the participants with a positive serological result. All ELISA-S-positive participants had anti-SARS-CoV-2 antibodies that stemmed either from a history of COVID-19 infection or vaccination and/or both. For quantitative and seroneutralization analyses, we considered an unvaccinated participant with or without a known history of infection as a “natural infection” case because he or she had anti-SARS-CoV-2 antibodies. We also described the participants’ ELISA-S antibody levels, in order to follow the evolution of the rate of seroconversion over time. The *t*-test was used to compare antibody levels on ELISA-S in participants with antibody loss. The Kruskal–Wallis rank sum test was used to assess statistically significant differences in antibody levels according to the number of COVID-19 vaccine doses. Statistical significance was set at *p* < 0.05. The Spearman rank test was used for the analysis of correlations between the quantitative levels of anti-SARS-CoV-2 S IgG and neutralizing antibody titers. The results were interpreted as negligible (<0 to 0.19), weak (0.2–0.39), moderate (0.40–0.69), strong (0.70–0.89), or very strong (>0.9). All statistical analyses were performed using the R program, version 4.1.3 (R Foundation, Vienna, Austria) [20].

### 2.6. Ethics

All study activities were conducted in accordance with the ad hoc ethics committee (Personal Protection Committee #2020-A00711-38) and by the data protection officer of the University of Corsica. All study participants gave written informed consent before their enrollment into the study.

## 3. Results

### 3.1. Sociodemographic Data Description of the Study Population

A total of 295 persons (223 staff members and 72 students) participated in this three-phase serological follow-up that took place from 23 November 2020 to 31 July 2021. The main characteristics of the participants are summarized in Table 1.

Overall, 67.5% of the participants were women (n = 199) and the median participant age was 37 (range, 17–64) years. Last, 32.9% (n = 97) of the participants had at least one chronic disease (Table 1).

### 3.2. SARS-CoV-2 Infection History of the Study Population

The clinical characteristics and the serological status of the participants are presented in Table 2.

No participants were vaccinated in the first phase while 63.1% (n = 186) of the participants were received at least one dose of vaccine in the third phase (*p* < 0.001).

In phase 1, 95.0% of the participants (n = 280) were considered as being “Unvaccinated with no known history of infection”, whereas 5.0% (n = 15) were considered as being “Unvaccinated with a known history of infection”.

In phase 2, 80.3% of the participants (n = 237) were “Unvaccinated with no known history of infection”. A slight increase in “Unvaccinated with known infection history” participants was observed, from 5.0% (n = 15) in phase 1 to 13.6% (n = 40) in phase 2. In addition, 6.1% of the participants started to be “Vaccinated with no known history of infection” (n = 18).

In phase 3, 63.1% of the participants were vaccinated (n = 186) with at least one dose of vaccine and an increase in the number of participants with a known history of infection was evidenced, from 13.6% (n = 40) in the second phase to 16.6% (n = 49) in the third phase.

### 3.3. Seroprevalence of IgG Antibodies against the SARS-CoV-2 S Protein

Table 2 also shows the proportion of participants with a positive ELISA result for IgG antibodies against the SARS-CoV-2 S protein. Among the 295 participants, 34 were tested positive for antibodies against the SARS-CoV-2 S protein during the first phase (23 November 2020 to 31 January 2021), with a prevalence rate of 11.5%. During the second phase (1 March to 9 May 2021), 31 participants were tested positive for antibodies against the SARS-CoV-2 S protein, with a prevalence rate of 10.5%. Finally, 201 individuals were tested positive for SARS-CoV-2 S-protein antibodies, with a prevalence rate of 68.1%, in the final phase (14 June to 31 July 2021).

### 3.4. Description of ELISA-S Serological Results According to the Immune Status of the Participants

The ELISA-S serology results according to the immune status of the participants are reported in Table 3.

In phase 1, among the 280 participants who were defined as being “Unvaccinated with no known history of infection”, 92.1% (n = 258) of the participants had a negative ELISA-S serology result (Table 3). In contrast, 80% (n = 12 out of 15) of the participants who were defined as being “Unvaccinated with a known history of infection” had a positive ELISA-S serology result. Moreover, 7.9% (n = 22 out of 280) of the unvaccinated participants with no known history of infection showed antibodies against the SARS-CoV-2 S protein, whereas 20.0% (n = 3 out of 15) of the unvaccinated participants with a known history of infection did not show antibodies against the SARS-CoV-2 S protein.

In phase 2, 99.6% of the unvaccinated participants with no known history of infection (n = 236 out of 237) had a negative ELISA-S serological result. In contrast, 47.5% of the unvaccinated participants with a known history of infection (n = 19 out of 40) had a positive ELISA-S serology result. Finally, 61.1% (n = 11 out of 18) of participants vaccinated with at least 1 dose had positive ELISA-S serology result (Table 3). Moreover, 38.9% of the vaccinated participants with no known history of infection (n = 7 out of 18) did not show antibodies against the SARS-CoV-2 S protein at that time of the study.

In phase 3, 95.2% of the unvaccinated participants with no known history of infection (n = 79 out of 83) had a negative ELISA-S serology result. In contrast, 69.2% of the unvaccinated participants with a known history of infection (n = 18 out of 26) had a positive ELISA-S serology result. Finally, 96.3% and 100.0% of all participants vaccinated without or with a known history of infection had a positive ELISA-S serology result (n = 157 out of 163; and n = 23 out of 23, respectively) (Table 3).

### 3.5. Description of the Participants Who Lost Their Antibodies over Time

A total of 5.8% (n = 17 of 295) of the participants lost their antibodies between the first and second phase of the study period. Their median age was 38 (range, 17–60) years, and 76% were female (n = 13). Moreover, 29% (n = 5) of these participants had a chronic medical condition. The mean time between the first and second sampling ranged from 90 days. At the time of investigation, no neutralizing antibodies were detected in any of these individuals. We also compared the antibody level of these participants who lost their antibodies to those who did not; the mean antibody level was 86.64 and 44.32 BAU/mL, respectively (*p* = 0.001). No participants lost these antibodies between the second and third phases of the study.

### 3.6. Comparisons of IgG Antibody Levels against the SARS-CoV-2 S Protein According to Participants’ Immune Status

**Figure 3:**
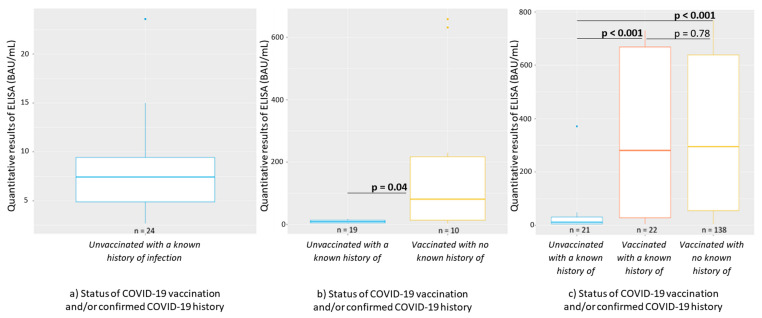
Levels of the IgG antibodies (BAU/mL) against the SARS-CoV-2 S protein by participants’ immune status according to (**a**) phase 1; (**b**) phase 2; (**c**) phase 3 of the study. The line in the center of the box depicts the median; the lower and upper hinges correspond to the first and third quartiles; and the distance between the first and third quartiles corresponds to the interquartile range.

**Figure 4:**
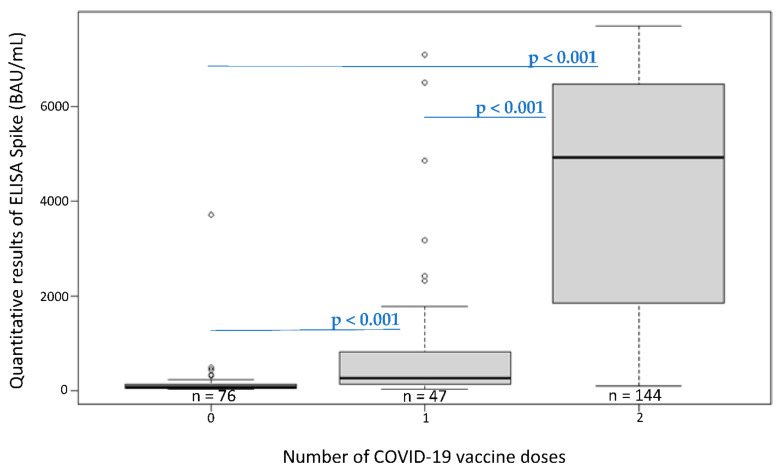
Levels of the IgG antibodies (BAU/mL) of participants with a quantitative ELISA-S result at the time of phase 3 according to their number of COVID-19 vaccine doses.

The participants’ quantification results of IgG antibody levels against the SARS-CoV-2 S protein and the participants according to their immune status are presented in Table 4, Figure 3 and Figure 4.

The line in the center of the box depicts the median; the lower and upper hinges correspond to the first and third quartiles; and the distance between the first and third quartiles corresponds to the interquartile range. The Kruskal–Wallis rank sum test was used to assess statistically significant differences in antibody levels according to the number of COVID-19 vaccine doses.

We observed a low amount of antibodies among unvaccinated participants with a known history of infection at the time of phases 1 and 2, from 86.6 to 88.2 BAU/mL, respectively (Table 4 and Figure 3). However, we detected higher antibody titers among vaccinated participants, with an increase in the amount of antibodies observed between phases 2 and 3 for participants who were vaccinated with no known history of infection, from 1909.3 to 3402.9 BAU/mL, respectively. In fact, an increase in the amount of antibodies was observed (all phases combined) when participants received a new dose of vaccine: 0 doses (166 BAU/mL) vs. 1 dose (934 BAU/mL); and 1 dose (934 BAU/mL) vs. 2 doses (4180 BAU/mL) (*p* < 0.001; Figure 4). In addition, we observed a slight increase in the antibody amount of antibodies when participants who were vaccinated had a known history of infection, with an average quantification of 3593.8 BAU/mL, compared with participants who were vaccinated with no known history of infection, with an average quantification of 3402.9 BAU/mL (Table 4 and Figure 3).

Furthermore, the group of participants with chronic diseases was treated separately from the other participants to observe whether they were likely to influence immune responses. No significant differences were found between these two groups.

### 3.7. Description of the Seroneutralization Results According to the Immune Status of the Participants and Correlation with ELISA Test Results

The seroneutralization results according to immune status are listed in Table 5.

In phase 1, 34 participants had a positive ELISA-S serological result and all of whom were grouped in the “unvaccinated with a known history of infection” category. Of these 34 participants, 29.4% (n = 10) had a positive seroneutralization result (Table 5).

In phase 2, 31 participants had a positive ELISA-S serological result. Of these 31 participants, 83.9% were tested by seroneutralization (n = 26). Overall, 84.6% of the participants (n = 22 out of 26) had a positive seroneutralization result. Of these 22 participants, 68.2% (n = 15) were in the “unvaccinated with a known history of infection” group and 31.8% (n = 7) were vaccinated with no known history of infection. In total, approximately 80% of the participants in these two categories had a positive seroneutralization result.

In phase 3, 201 participants had a positive ELISA-S serological result. Of these 201 participants, 98.0% (n = 197) were tested by seroneutralization. Overall, 88.8% (n = 175 out of 197) of the participants had a positive seroneutralization result. Of these 175 participants, 7.4% (n = 13) were in the “unvaccinated with a known history of infection” group, 80.6% (n = 141) were “vaccinated with no known history of infection”, and 12.0% (n = 21) were “vaccinated with a known history of infection”. In total, 65% (n = 13 out of 20) of the participants with an “unvaccinated with a known history of infection” status had a positive seroneutralization result. Finally, seroneutralization was greater in vaccinated participants with or without a known history of infection, with approximately 91% of the vaccinated participants without or with a known history of infection having a positive seroneutralization result compared with 65% of unvaccinated participants with a known history of infection (Table 5).

**Figure 5:**
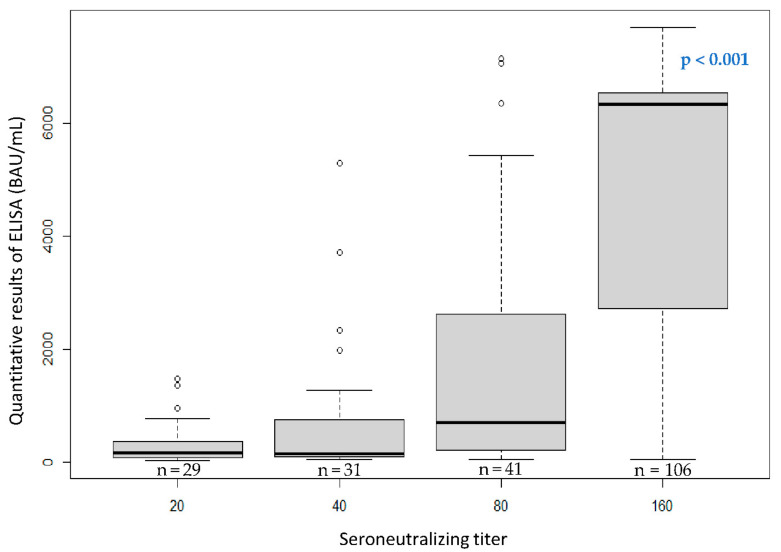
Boxplot of the quantitative ELISA-S results according to neutralizing antibody titers among the participants.

The quantitative ELISA-S results according to neutralizing antibody titers among the participants are presented in Figure 5.

Finally, the anti-S protein IgG antibody titers were strongly and positively correlated to the neutralizing antibody titers (Spearman’s rank correlation coefficient, 0.71), with statistical significance (*p* < 0.001; Figure 5).

## 4. Discussion

The findings of this study, which included serum samples from 295 participants collected from 23 November 2020 to 31 July 2021 and covering 3 distinct time periods during the SARS-CoV-2 pandemic, are presented here. The first study took place from 23 November 2020 to 31 January 2021 [18], occurring between the second and third epidemic wave of COVID-19, and showed a seroprevalence of 11.5%. The follow-up study took place from 1 March 2021 to 9 May 2021 for phase 2, and from 14 June 2021 to 31 July 2021 for phase 3, occurring in the third epidemic wave for phase 2 and the fourth epidemic wave of COVID-19 for phase 3, respectively. In this study, we characterized the temporal changes in the anti-SARS-CoV-2 IgG antibody levels in the participants. The results demonstrated: (i) a slight decrease in seroprevalence between phase 1 and 2, from 11.5% to 10.5%, respectively, and (ii) an increase in seroprevalence between phases 2 and 3 from 10.5% to 68.1%, respectively. In addition, the French national vaccination campaigns started at the end of 2020 with a priority for health-care workers, fragile people on 4 January 2021, then for all volunteers >18 years of age starting on 15 June 2020 [21]. This also reflects the increase in seroprevalence during the third phase, which occurred mostly among those who were vaccinated. However, in the absence of a vaccine, the seroprevalence measured during the first phase reflected viral infections only.

In fact, seroprevalence surveillance and knowledge about humoral responses are essential to guide vaccination strategies in population. However, few data are available on the durability of humoral responses against SARS-CoV-2 over a long period [9,14,22,23,24]. Our results demonstrated that some participants lost their natural antibodies between phase 1 and phase 2 of the study with a delay of approximately 3 months between the first and second sampling. These results are in agreement with previous studies showing a decrease in natural antibody levels among infected and convalescent individuals after 2 to 4 months, and with significantly reduced levels of anti-SARS-CoV-2 [14,25,26]. In our study, participants with a known history of infection and without vaccination exhibited relatively low antibody titers. This finding demonstrated that a natural infection without vaccination does not confer effective protection against SARS-CoV-2. Importantly, natural IgG antibody levels remained stable for 5 months between phase 1, with a mean of 86.6 BAU/mL, and phase 2, with a mean of 88.2 BAU/mL, for unvaccinated participants with a known history of infection. It was previously reported that the titers anti-SARS-CoV-2 remained stable for 5 months after infection [4]. In addition, previous studies have investigated the long-term humoral immune response in naive and previously infected volunteers who received SPUTNIK V vaccine, demonstrating that antibody titers following vaccination began to decrease after 60 days post-vaccination, remained detectable at 90 days, and persisted at 180 days [27].

Furthermore, we analyzed the effects of vaccination on antibody levels. Several vaccinated individuals did not have IgG antibodies at the time of the study because of the short delay between the date of the vaccine injection and the date of the blood collection (between 3 and 26 days). With the exception of these few individuals, vaccinated participants with no known history of infection showed antibody levels increased by a factor 2 between phases 2 and 3. One explanation for this finding may be that, between the second and third phases, the participants received a new dose of vaccine, which increased antibody titers. This is consistent with another study that showed that, even after only the first vaccine dose, the levels of IgG generated are substantial [14,27]. Interestingly, the evidence showed that the immune response was stronger in those with a known history of infection than it was in those with no known history of infection, regardless of vaccination status. This is in line with previous studies [28,29] that reported a significant increase in antibody levels in previously infected individuals. Importantly, the IgG antibody levels after the first dose of vaccine were approximately 22 times higher than those of a simple natural infection. As observed in previous studies [27,30,31], we could confirm that vaccination leads to a strong increase in the amount of antibodies. Consistent with the findings of the clinical trials [32,33,34], we found that individuals who received two vaccine doses had higher antibody levels than unvaccinated individuals or those who received a single vaccine dose. In addition, we found a slight increase in the amount of antibodies in vaccinated participants with a known history of infection compared to vaccinated participants with no known history of infection. Previous studies [17,35,36] have shown that vaccinated individuals who experienced COVID-19 developed more antibodies, in contrast to vaccinated individuals who did not. This striking variation in the rates of seroconversion may account for the lower incidence of breakthrough infection previously described in this particular population [35]. This point reinforces the single-dose strategy for vaccinated individuals with previous COVID-19 infection.

The determination of neutralizing antibodies to anti-SARS-CoV-2 antibodies is essential for understanding the possible protective effects of the immune response. Effectively, seroconversion of neutralizing antibodies in individuals with mild COVID-19 may take longer than in those with severe COVID-19 [23]. Studies have reported that 50% to 100% of patients with confirmed COVID-19 seroconverted to have neutralizing antibodies. Our analysis indicated similar rates of neutralizing antibodies and showed also that vaccinated participants with or without a known history of infection were more likely to seroneutralize than those unvaccinated with a known history of infection. This is in agreement with another study, in which most plasmas obtained from individuals recovering from COVID-19 did not contain high levels of neutralizing activity [37]. In contrast to the finding that vaccination induces the production of abundant neutralizing antibodies, this has been confirmed by a previous study [38]. The determination of the immune correlates of protection against SARS-CoV-2 is necessary to predict the decrease in antibodies and determine vaccination strategies. In agreement with previous studies [4,10,39], we found a strong positive correlation between anti-S and neutralizing antibodies.

The main strength of this study was that it evaluated seropositivity during an eight-months follow-up, thus allowing the determination of the evolution of anti-SARS-CoV-2 IgG antibodies. Furthermore, the use of two serological tests among participants (ELISA-S, and seroneutralization), and the quantification of anti-SARS-CoV-2 IgG antibodies and neutralization titers, were other strengths of this study. Nevertheless, our study also had several limitations. First, it is possible that the participants are not representative of the population as a whole, thus limiting the generalizability of our findings. Second, we relied on self-reported results of SARS-CoV-2 infection, which may have introduced a bias in the reporting of the results. Third, in order to use the same methodology as before, we could not measure antibodies against the N protein of SARS-CoV-2, and all previous asymptomatic cases were certainly not detected; this may lead to misclassification in the pre-exposure groups. Lastly, we analyzed the humoral immune response, but we could not evaluate the cellular immune response.

## 5. Conclusions

The results reported here primarily reflect the circulation of SARS-CoV-2 among the participants from November 2020, during the second wave in France and at the start of the COVID-19 vaccination campaign, to the end of July, at the time of the fourth wave. We assessed the dynamics of antibody responses in participants as a function of their immune status. The results of our study demonstrated that single infection does not confer effective protection against SARS-CoV-2. Our results support vaccination because of the rapid decline in antibody titers after a single infection. In individuals with a known history of SARS-CoV-2 infection, two doses of vaccine provide effective coverage. The timing of subsequent booster doses still needs to be determined.

Our findings, together with others, indicate the need to monitor the antibodies of individuals to inform the development of future vaccination strategies.

## Figures and Tables

**Table 1:** Sociodemographic characteristics of the study population.

Characteristic	Overall n = 295 ^1^
**Median age (min; max)**	37 (17; 64)
**Age group**	
<20 years	14 (4.7%)
20–29 years	92 (31.2%)
30–39 years	70 (23.7%)
40–49 years	64 (21.7%)
>50 years	55 (18.6%)
**Gender**	
Female	199 (67.5%)
Chronic diseases *	97 (32.9%)
NA **	1

^1^ Mean/Median (min–max); n (%). * Chronic diseases (obesity, diabetes, hypertension, heart disease, asthma, pulmonary pathology, liver disease, chronic neurological disease, chronic renal failure, rheumatological disease, cancer, and immunosuppressive disease). ** NA means “Not applicable”, corresponding to missing data.

**Table 2:** Clinical characteristics of the study population.

Characteristic	Phase 1	Phase 2	Phase 3	*p* *
	(23 November 2020 to 31 January 2021),	(1 March 2021 to 9 May 2021),	(14 June 2021 to 31 July 2021),	
	n = 295	n = 295	n = 295	
**Evolution of COVID-19 vaccine coverage at each phase**				**<0.001**
0 doses	295 (100.0%)	277 (93.9%)	109 (36.9%)	
1 dose	0 (0%)	13 (4.4%)	47 (15.9%)	
2 doses	0 (0%)	5 (1.7%)	139 (47.2%)	
**COVID-19 vaccination and/or confirmed history of COVID-19**				**<0.001**
Unvaccinated with no known history of infection	280 (95.0%)	237 (80.3%)	83 (28.2%)	
Unvaccinated with known history of infection	15 (5.0%)	40 (13.6%)	26 (8.8%)	
Vaccinated with no known history of infection	0 (0%)	18 (6.1%)	163 (55.2%)	
Vaccinated with known history of infection	0 (0%)	0 (0%)	23 (7.8%)	
**Serological status (ELISA-S)**				**<0.001**
Positive	34 (11.5%)	31 (10.5%)	201 (68.1%)	
Negative	261 (88.5%)	264 (89.5%)	94 (31.9%)	

* The *p*-value compares the results obtained among the three phases of the study.

**Table 3:** ELISA-S results according to participants’ immune status.

Overall (N = 295)	Phase 1 (23 November 2020 to 31 January 2021)		Phase 2 (1 March 2021 to 9 May 2021)		Phase 3 (14 June 2021 to 31 July 2021)	
	Positive ELISA-S Serological Result n/N % [95% CI]	Negative ELISA-S Serological Result n/N % [95% CI]	Positive ELISA-S Serological Result n/N % [95% CI]	Negative ELISA-S Serological Result n/N % [95% CI]	Positive ELISA-S Serological Result n/N % [95% CI]	Negative ELISA-S Serological Result n/N % [95% CI]
Total (All Groups Combined)	34/295 11.5% [8.2–15.9%]	261/295 88.5% [84.1–91.8%]	31/295 10.5% [7.4–14.7%]	264/295 89.5% [85.3–92.6%]	202/295 68.5% [62.8–73.7%]	93/295 31.5% [26.3–37.2]
Unvaccinated with no known history of infection	22/280 7.9% [5.1–11.8%]	258/280 92.1% [88.2–94.9%]	1/237 0.4% [0.02–2.7%]	236/237 99.6% [97.3–100%]	4/83 4.8% [1.6–12.5%]	79/83 95.2% [87.5–98.4]
Unvaccinated with known history of infection	12/15 80.0% [51.4–94.7%]	3/15 20.0% [5.3–48.6%]	19/40 47.5% [31.8–63.7%]	21/40 52.5% [36.3–68.2%]	18/26 69.2% [48.1–84.9%]	8/26 30.8% [15.1–51.9]
Vaccinated with no known history of infection	~	~	11/18 61.1% [36.1–81.7%]	7/18 38.9% [18.3–63.9]	157/163 96.3% [91.8–98.5%]	6/163 3.7% [1.5–8.2]
Vaccinated with known history of infection	~	~	~	~	23/23 100.0% [82.2–100]	0/23 0.0% [0–17.8]

~ means no data in this category.

**Table 4:** Comparisons of IgG antibody levels to SARS-CoV-2 S protein according to participants’ immune status.

**Total (All Groups Combined)**	**Phase 1** **(23 November 2020 to 31 January 2021)** **(n = 34)**		**Phase 2** **(1 March 2021 to 9 May 2021)** **(n = 31)**		**Phase 3** **(14 June 2021 to 31 July 2021)** **(n = 201)**	
	**Mean (BAU/mL) [95% CI]** **(Min–Max)**	**Effective (n)**	**Mean (BAU/mL) [95% CI]** **(Min–Max)**	**Effective (n)**	**Mean (BAU/mL) [95% CI]** **(Min–Max)**	**Effective (n)**
	**86.6 [62.9–110.3]** **(26.5; 235.9)**	**24**	**716.1 [76.7–1355.6]** **(26.3; 6585.6)**	**29**	**3069.3 [2660.0–3478.6]** **(27.6; 7680.0)**	**181**
Unvaccinated with a known history of infection	86.6 [62.9–110.3] (26.5; 235.9)	24	88.2 [63.6–112.8] (26.3; 169.5)	19	327.8 [–30.7–686.3] (27.6; 3712.0)	21
Vaccinated with no known history of infection	~	~	1909.3 [103.5–3715.1] (28.2; 6585.6)	10	3402.9 [2945.4–3860.4] (42.9; 7680.0)	138
Vaccinated with a known history of infection	~	~	~	~	3593.8 [2232.1–4955.4] (37.6; 7296.0)	22
NA *		10		2		20

* NA means “Not applicable”, corresponding to missing data. ~ means no data in this category.

**Table 5:** Seroneutralization results of the participants according to their immune status.

	Phase 1 (23 November 2020 to 31 January 2021) (n = 34)		Phase 2 (1 March 2021 to 9 May 2021) (n = 31)		Phase 3 (14 June 2021 to 31 July 2021) (n = 201)	
**Total (All Groups Combined)**	**Positive** **Seroneutralization** **Result** **(n/N)** **% [95% CI]**	**Negative** **Seroneutralization** **Result** **(n/N)** **% [95% CI]**	**Positive** **Seroneutralization** **Result** **(n/N)** **% [95% CI]**	**Negative** **Seroneutralization** **Result** **(n/N)** **% [95% CI]**	**Positive** **Seroneutralization** **Result** **(n/N)** **% [95% CI]**	**Negative** **Seroneutralization** **Result** **(n/N)** **% [95% CI]**
	**10/34** **29.4% [15.7–47.7]**	**24/34** **70.6% [52.3–84.3]**	**22/26** **84.6% [64.3–95.0]**	**4/26** **15.4% [5.05–35.7]**	**175/197** **88.8% [83.4–92.7]**	**22/197** **11.2% [7.28–16.6]**
Unvaccinated with a known history of infection	10/34 29.4% [15.7–47.7]	24/34 70.6% [52.3–84.3]	15/18 83.3% [57.7–95.6]	3/18 16.7% [4.41–42.3]	13/20 65.0% [40.9–83.7]	7/20 35.0% [16.3–59.1]
Vaccinated with no known history of infection	~	~	7/8 87.5% [46.7–99.3]	1/8 12.5% [0.66–53.3]	141/154 91.6% [85.7–95.2]	13/154 8.44% [4.76–14.3]
Vaccinated with a known history of infection	~	~	~	~	21/23 91.3% [70.5–98.5]	2/23 8.70% [1.52–29.5]
NA *	0	0	5	5	4	4

* NA means “Not applicable”, corresponding to missing data. ~ means no data in this category.

## Data Availability

Not applicable.
